# A New Integrated Variable Based on Thermometry, Actimetry and Body Position (TAP) to Evaluate Circadian System Status in Humans

**DOI:** 10.1371/journal.pcbi.1000996

**Published:** 2010-11-11

**Authors:** Elisabet Ortiz-Tudela, Antonio Martinez-Nicolas, Manuel Campos, María Ángeles Rol, Juan Antonio Madrid

**Affiliations:** 1 Chronobiology Laboratory, Department of Physiology, University of Murcia, Murcia, Spain; 2 Department of Computer Science and Systems, University of Murcia, Murcia, Spain; University of California San Diego, United States of America

## Abstract

The disruption of the circadian system in humans has been associated with the development of chronic illnesses and the worsening of pre-existing pathologies. Therefore, the assessment of human circadian system function under free living conditions using non-invasive techniques needs further research. Traditionally, overt rhythms such as activity and body temperature have been analyzed separately; however, a comprehensive index could reduce individual recording artifacts. Thus, a new variable (TAP), based on the integrated analysis of three simultaneous recordings: skin wrist temperature (T), motor activity (A) and body position (P) has been developed. Furthermore, we also tested the reliability of a single numerical index, the Circadian Function Index (CFI), to determine the circadian robustness. An actimeter and a temperature sensor were placed on the arm and wrist of the non-dominant hand, respectively, of 49 healthy young volunteers for a period of one week. T, A and P values were normalized for each subject. A non-parametric analysis was applied to both TAP and the separate variables to calculate their interdaily stability, intradaily variability and relative amplitude, and these values were then used for the CFI calculation. Modeling analyses were performed in order to determine TAP and CFI reliability. Each variable (T, A, P or TAP) was independently correlated with rest-activity logs kept by the volunteers. The highest correlation (r = −0.993, p<0.0001), along with highest specificity (0.870), sensitivity (0.740) and accuracy (0.904), were obtained when rest-activity records were compared to TAP. Furthermore, the CFI proved to be very sensitive to changes in circadian robustness. Our results demonstrate that the integrated TAP variable and the CFI calculation are powerful methods to assess circadian system status, improving sensitivity, specificity and accuracy in differentiating activity from rest over the analysis of wrist temperature, body position or activity alone.

## Author Summary

Faced with environmental cycles and daily alternation between light and darkness, organisms have evolved a time measuring mechanism, the biological clocks. Besides following circadian rhythms, all physiological variables must be coordinated with one another, like an orchestra led by a conductor; if the appropriate rhythm is not kept, noise rather than music is produced. In an organism, when this temporal order is disrupted due to aging or shift work, health is compromised. Afflictions include metabolic syndrome, diabetes and cardiovascular diseases, among others, or even worse prognosis of preexisting illnesses like cancer. Since the circadian pacemaker (suprachiasmatic nuclei) is located deep within the brain in humans, the only way to evaluate its function is by assessing the output signals, observing marker rhythms such as the sleep-wake cycle, body temperature or activity. The problem is that isolated variable measurement is not error free. However, we can increase reliability by combining the information from several circadian marker rhythms in an integrated variable that we have called TAP (Temperature, Activity and Position), a methodological approach that has not been used before, that in conjunction with a new index called Circadian Function Index, provides a useful tool for standardizing the status of the circadian system.

## Introduction

Circadian system disruption in humans has been associated with the development of chronic illnesses and worsening of pre-existing conditions such as cancer, premature ageing, metabolic syndrome, cardiovascular diseases, cognitive impairment and mood disorders (for a review, see [1]). Therefore, proper assessment of the circadian system function under normal living conditions using non-invasive techniques is a current issue in need of further research [2]–[5].

The circadian system consists of a set of structures involved in the generation of circadian rhythms in behavioral, physiological and biochemical variables, as well as in the external and internal synchronization of these variables to environmental cues and to each other, respectively. Three different approaches have been developed to determine circadian system function in humans. Many researchers have measured the output of the hypothalamus's major circadian clock, the suprachiasmatic nuclei (SCN), after trying to eliminate all influence from the external factors (masking factors) that may affect the variable being measured. One example may be the use of constant 48-h routine protocols in which the subjects were kept under constant light, temperature and body position, and frequently fed isocaloric snacks [6], or the forced desynchronization of the internal time clock by forcing individuals to live according to sleep-wake cycles outside their entrainment limits, i.e., 28h or 20h [7]. A second approach consists of recording rhythmic variables in subjects under controlled environmental conditions, after which it becomes necessary to use mathematical procedures in a demasking process to eliminate the effect of the rhythmic environment [8], [9]. Finally, the third approach is based on recording circadian marker rhythms in subjects under normal living conditions, with the underlying assumption that masking and clock-controlled processes are both important to allow humans to cope with environmental rhythmic challenges [10]. This last approach is the only way to assess the functionality of the circadian system in humans over long periods of time under normal living conditions.

Theoretically, most overt rhythms controlled by the circadian clock, which can be measured easily and with minimal subject discomfort, can be used as marker rhythms to assess circadian system function [2]. In practice, the most widely used rhythms are melatonin, cortisol, core body temperature and rest-activity cycles [11], [12]. The melatonin rhythm is considered to be one of the most reliable marker rhythms; however, its measurement is time consuming, and plasma or saliva sampling requires an intravenous catheter or the subject's active collaboration, respectively. Moreover, the melatonin rhythm in subjects under normal living conditions can be masked by a number of factors including posture, exercise, sleep or sleep deprivation, caffeine, certain drugs, such as beta-blockers and NSAIDS, and, in particular, nocturnal light exposure [13]. Cortisol is also used as a marker rhythm; however, it is subjected to ultradian rhythmicity and is masked by many factors, such as physical exercise, physiological stress, lighting conditions, the sleep-wake cycle and high-protein intake [14]–[16].

Core body temperature rhythm (CBT) is frequently used as a circadian marker rhythm because it is relatively easy to record and the data can be analysed immediately. However, the most frequent way to measure CBT, rectal temperature, causes subject discomfort. Recently, eatable telemetric pills are also available for core temperature recordings. Nevertheless, the recordings are limited by the duration and dependent on intestinal transit. In addition, the rhythm is also masked by factors like posture, physical activity, meals, environmental light and temperature in both cases [2].

Rest-activity rhythm measurement by actimetry is a simple, non-invasive method for indirectly evaluating the sleep-wake cycle. Therefore, it can be considered a marker rhythm. But as occurs with other methods, actimetry is subjected to masking and artifacts, such as difficulties related to differentiating between the onset of nocturnal rest and sensor removal for bathing before going to bed, bed partner movements, sleeping when travelling in a car or train, etc. [17], [18]

Recently, our group proposed wrist skin temperature as a possible alternative method for evaluating circadian system status in humans under normal living conditions [10]. This rhythm is in part the result of an alternating balance between parasympathetic (vasodilation) and sympathetic (vasoconstriction) actions on peripheral skin vessels, driven by the SCN [19]–[21]. Wrist skin temperature increases during rest periods associated with sleep and decreases during activity periods in proportion to the level of arousal [10]. Again, the existence of masking factors such as environmental temperature and posture reduces its accuracy when used by itself to evaluate circadian function.

The existence of artifacts and different masking factors for all the rhythmic variables considered to be circadian markers led us to propose the use of a combination of three rhythmic variables for ambulatory monitoring. Therefore, we propose, for the first time, to integrate skin temperature, along with actimetry and body position data into a single variable to evaluate the status of the human circadian system under normal living conditions. This simple, non-invasive and practical approach will encourage clinicians to treat patients with circadian rhythm disorders and facilitate chronotherapy individualization.

## Methods

### Study population

Forty-nine subjects volunteered for this study. They included 25 women and 24 men ranging from eighteen to forty years of age (21.30±4.44). All participants received appropriate information about the study characteristics and signed an informed consent form before their inclusion in the study. The study was approved by the Ethics Committee of the University of Murcia.

Participants were recruited from among Biology and Medical students. They were all healthy and presented no physical conditions that disturbed their sleep (e.g. sleep apnea, asthma, periodic limb movement, diabetes, etc.). Furthermore, they were encouraged to maintain their normal life style during the week of the study and were monitored under free-living conditions.

### Rest and food schedules

Throughout the study, all subjects were instructed to keep a sleep and food diary designed by the Chronobiology Lab at the University of Murcia [10]. Participants were instructed to log the following on a daily basis: the time they went to bed, the time of lights off, nocturnal awakenings lasting more that 10 min, sleep offset, the time they woke up, the time and duration of naps and the time of onset for the three main meals.

### Temperature rhythm

**Figure 1:**
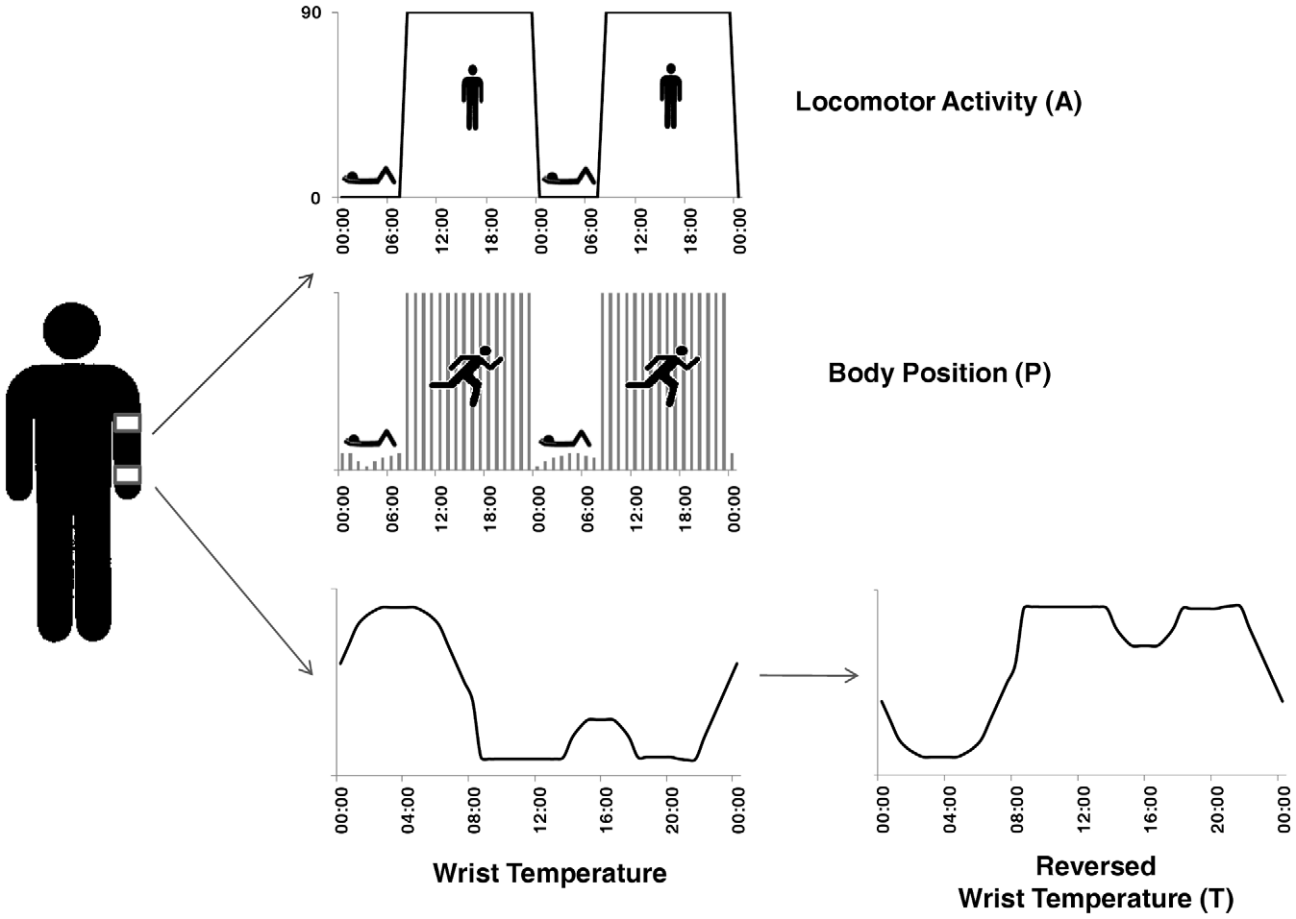
Body location of all sensors and simplistic representations of variables extracted. The activity data logger is placed in a sport band, on the upper non-dominant arm and the temperature data logger on the wrist of the non-dominant hand. Here it is shown the three variables selected for the study and a simplistic representation of their behaviour. Motor activity and body position show their higher levels during day-time, when the subject is active. Wrist temperature (T) profile, on the contrary, show its higher levels during the night, when the subject is resting. Then, to calculate TAP, we reversed T profile.

The wrist temperature rhythm was assessed continuously for 7 days using a temperature sensor (Thermochron iButton DS1921H, Dallas, Maxim) with a sensitivity of 0.1°C and programmed to sample every 10 minutes. It was attached to a double-sided cotton sport wrist band, and the sensor surface was placed over the inside of the wrist on the radial artery of the non-dominant hand (Figure 1), as previously described [10].

### Body position and rest-activity rhythm

The body position and rest-activity rhythm was assessed over the same 7 days using an actimeter (Hobo Pendant G Acceleration Data Logger, Massachusetts, USA) placed on the non-dominant arm by means of a sports band, with its X-axis parallel to the humerus bone (Figure 1). This actimeter was a three-channel logger with 8-bit resolution that can record up to 21,800 combined X-, Y-, and Z-axis acceleration and static position readings or internal logger events. This particular logger uses an internal three-axis accelerometer with a range of ±3g based on micro-machined silicon sensors consisting of beams that deflect with acceleration. The sensor dimensions are 58×33×23 mm and it weighs 18g.

The sensor was programmed to record data every 30 seconds. The information stored in the actimeter was transferred through an optical USB Base Station (MAN-BASE-U-4, HOBO) to a personal computer using the software provided by the manufacturer (HOBOware 2.2).

From the information provided by the actimeter, we defined 2 variables: motor activity (A) and body position (P). Motor activity, expressed as degrees of change in position, was calculated at 30-s intervals as the sum of the first derivative of the angle formed between the current sensor position and its position 30 s before, taking into account the X, Y and Z axes. Body position was calculated as the angle between the X-axis of the actimeter and a horizontal plane. Thus, P oscillated between 0° for maximum horizontality and 90° for maximum verticality.

### Data analysis

Since no marked differences by gender were detected (Student's t test), male and female data were pooled and analysed together (note that the number of male and female was balanced).

### Data processing

Firstly, data were filtered in order to eliminate erroneous measurements, such as those produced by temporarily removing the sensors. In order to obtain the same sampling frequency for all variables for the purpose of computing TAP values, motor activity and body position data were added up and averaged, respectively, in 10-minute intervals (i.e., the sampling rate of wrist temperature). However, motor activity was expressed as degrees of position change per minute by dividing these previous values by 10.

Rest log data were converted into a binary code, in which 1 corresponded to a declared resting period and 0 to an activity period.

### TAP

In order to obtain TAP we first normalized the 3 variables (T, A and P) by calculating the 95^th^ and 5^th^ percentiles for each variable and volunteer. In a typical wrist temperature rhythm, the highest values are seen at night when the subject is asleep and the lowest values during the day when the subject is awake, whereas the opposite occurs in the case of motor activity and body position. Normalized wrist temperature values were therefore inverted, so that the maximum values for all 3 variables occurred at the same time of the day.

In a third step, we calculated the mean of the 3 normalized variables. Thus, 0 corresponded to complete rest and sleep, and 1 to periods of high arousal and movement.

TAP calculation was performed by using the next formula:
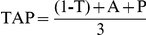



### Sensitivity, specificity and agreement rates

In order to objectively evaluate whether TAP improved the accuracy of rest-activity deduction as compared to each variable considered separately, we analyzed their correlation to rest probability calculated from the rest-activity diaries. In order to do so, we first had to determine the thresholds, for TAP and each separate variable, above and below which a subject was considered to be awake and resting, respectively. To that end, we calculated the different thresholds for each of 6 randomly chosen subjects by iteratively changing the threshold value in order to maximize the agreement rate between the prediction and the corresponding rest-activity diary. The values thus obtained for each variable were then averaged in order to calculate the final thresholds that were later applied to the whole group. If the value of a variable was above its threshold, it scored 0 (meaning awake), and if the value was under that threshold, it was assigned a score of 1 (resting). Furthermore, specificity, sensitivity and agreement rates were also calculated. Sensitivity reflected the probability of finding an actual resting individual when the TAP score indicated rest. On the contrary, specificity tried to find the probability of agreement when a subject was actually active and our TAP variable also scored a non-rest period. Finally, agreement rates represent the proportion of periods scored as “rest” by TAP that are truly “rest” periods based on the sleep logs analysis.
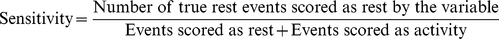


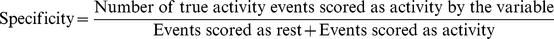


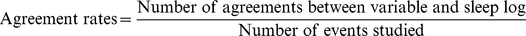
In order to check whether these parameters were statistically different between them obtained for every variable and TAP, we performed χ^2^ analysis.

### Correlations between variables

Linear regressions were performed for TAP and each single variable with respect to the rest periods indicated by the subjects. Significant differences in these correlations were established using a specific test (www.fon.hum.uva.nl/Service/Statistics/Two_Correlations.html).

### Temporal series analysis

In order to characterize the circadian pattern for TAP and each single variable, we performed a non-parametric analysis (as previously described [22]), including relative amplitude (RA), interdaily stability (IS), intradaily variability (IV), the mean value and timing of five consecutive hours with the lowest values (VL5 and L5 respectively) and the mean value and timing of ten consecutive hours with the highest values (VM10 and M10, respectively). IS quantified rhythm stability over different days. It varied between 0 for Gaussian noise, and 1 for a perfect stability, where the rhythm repeated itself exactly day after day. IV showed the fragmentation of the rhythm; its values oscillated between 0 (when the wave was perfectly sinusoidal) and 2 (Gaussian noise). RA referred to the difference between the VM10 and VL5, divided by VM10+VL5.

In addition, we generated weekly representations for all variables studied, as well as mean waveforms for every subject and the group as a whole.

Besides, in order to further analyse CFI, its scores were correlated to parametrical tests such as power content of the first harmonic and percentage of variance explained by the rhythm in a cosinor analysis, performed by the program El Temps (Diez Noguera, 1999).

### Circadian Function Index (CFI) configuration

CFI incorporates three parameters, IV, IS and RA, from the TAP variable. IV values were inverted and normalized between 0 and 1, with 0 being a noise signal, and 1 a perfect sinusoid. Finally, CFI was calculated as the average of these three parameters. Consequently, CFI oscillates between 0 (absence of circadian rhythmicity) and 1 (a robust circadian rhythm).

In this paper CFI has been calculated only for TAP. However, it can be calculated for other variables such as temperature, activity or position separately.

### TAP and CFI simulations

In order to determine whether the TAP and CFI accurately described circadian function, we performed a computational simulation of TAP with different levels of noise and instability in the rest-activity ratio. This kind of methodological approach allows verifying whether CFI varies as expected depending on noise and instability increases, as well as obtaining the corresponding waveform of TAP.

On the one hand, TAP was simulated from a squared wave, considering the activity phase as 66% of the total time of day. A kind of noise present in biological signals, the fractal noise was included in different percentages (from 0 to 100%) to simulate a continuous gradient between normal and extreme situations.

Secondly, we performed these same simulations for a sinusoidal waveform since many rhythms adjust to this kind of wave with two levels of noise (0 and 60%).

Finally, we introduced a new parameter in the simulations according to the instability in the ratio activity/rest for a squared waveform. We chose a 20% in instability for two percentages of noise, 0 and 60%.

Simulations were performed using the Syntesi program by Diez-Noguera (Barcelona, 2007).

## Results

**Figure 2:**
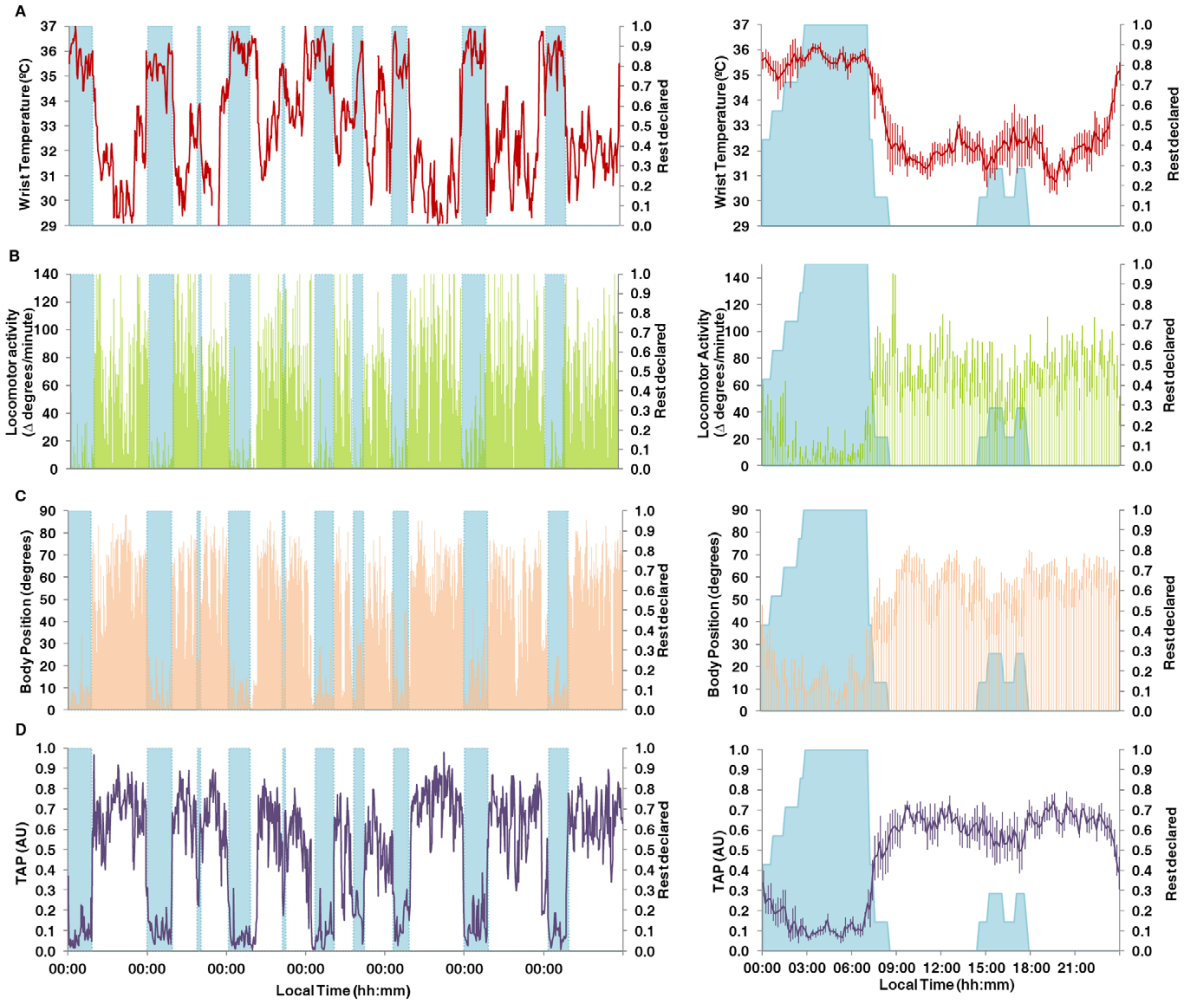
Individual weekly recording of all variables. Wrist temperature (A) in red, motor activity (B) in green, body position (C) in orange and TAP (D) in violet of a subject taken as an example, on the left. On the right it is represented the mean waveform of every variable for the same subject. Shaded blue areas coincide with sleep declared by subjects. On the right every variable is represented as value ± SEM.

A representative individual record from a normal-living subject is shown in Figure 2, which includes weekly recordings (on the left) and mean waveforms (on the right) for wrist temperature (2A), motor activity (2B), body position (2C) and TAP (2D). As expected, wrist temperature rose just before going to sleep (Figure 2A, right), remained elevated over night and decreased after waking. Note the close relationship between temperature increases and rest episodes, except on the third and fourth nights, when the high temperature period was longer than the rest period reported by the subject. As expected, motor activity (Figure 2B) displayed higher values during the day and lower values at night, when the subject was resting. Likewise, body position (Figure 2C) reached values close to 0° mainly at night, when the subject was resting and values close to 90° when the subject was active during the day. Again, the reported rest did not exactly match the low motor activity (Figure 2B) and horizontal body position (Figure 2C) periods during the third and fourth nights.

**Figure 3:**
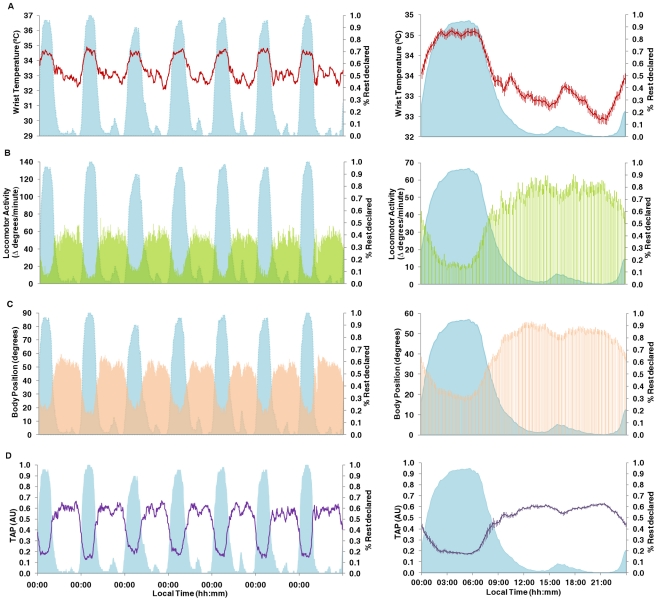
Complete week recording for every variable evaluated for the entire experimental group. On the left it is shown the weekly evolution of each variable and on the right its correspondent mean waveform. Wrist temperature (A) is represented in red, motor activity (B) in green, body position (C) in orange and TAP (D) in violet. On the right, every point is represented as value ± SEM. Please note that scales vary in mean waveforms representations on the right and representations on the left lack of SEM for a better understanding.

The integrated TAP variable is shown in Figure 2D. Each rest period, whether diurnal or nocturnal, coincided with a series of very low TAP values, and implied the coexistence of low activity, horizontal position and high temperature; Figure 3 shows the averaged results of the entire group for all rhythmic variables; with weekly recordings shown on the left and the mean waveform on the right. As expected, the average mean waveforms had smaller amplitudes than the individual ones. However, the average pattern of all variables agreed with the individual recordings previously shown, with high values for motor activity (Figure 3B) and position (Figure 3C) and low temperature values (Figure 3A) during the day, and the opposite during the night. The wrist temperature mean waveform (Figure 3A, right), on the other hand, was characterized by a sharp increase before bedtime, a nocturnal steady state coinciding with the sleep period and a pronounced drop immediately after awakening. There was a secondary peak around afternoon, a period associated with naps, and a dip between 20:00–22:00 h, a period already known as the “wake maintenance zone”. An almost inverse pattern was observed for motor activity and body position; however, no negative relationship was observed between T and A or P, in the wake maintenance zone.

The weekly average TAP data (Figure 3D, left) clearly showed how rest periods do correlate with decreased TAP values, during both the day and at night. The TAP mean waveform exhibited a close inverse relationship with reported rest periods and was characterized by a broad dip during sleep time, and a consistent, but transient TAP dip around 16:00–17:00 h, following the subjects' usual lunch time and during their normal nap period. Maximum TAP values were observed between 12:00–14:00 h and 20:00–22:00 h. Again, TAP values started to decrease in advance of bedtime.

In order to characterize the rhythmic patterns presented here, a non-parametric analysis was performed, the results for which are provided in Table 1. All variables exhibited similar IS values and they all had a very low dispersion, which indicates a high degree of similarity among the subjects. IV values, on the other hand, were quite different depending on the variable. Motor activity presented the highest IV, indicating a high level of fragmentation with substantial variability between consecutive periods, whereas temperature showed the lowest value. Motor activity was also the variable that showed the highest RA.

**Table 1:** Non-parametric analysis for every measured variable and TAP.

	T	A	P	TAP
	mean ± sem	mean ± sem	mean ± sem	mean ± sem
**IS**	0.44±0.02	0.41±0.01	0.50±0.02	0.55±0.02
**IV**	0.19±0.01	0.74±0.01	0.29±0.02	0.23±0.01
**RA**	0.51±0.02	0.69±0.02	0.56±0.02	0.56±0.02
**L5 (hh:mm)**	4:16±0:13	4:10±0:09	4:49±0:23	4:15±0:09
**M10 (hh:mm)**	17:21±0:18	16:25±0:16	15:09±0:24	16:45±0:14
**VL5**	0.20±0.01	0.10±0.01	0.20±0.01	0.14±0.03
**VM10**	0.60±0.01	0.52±0.01	0.72±0.01	0.50±0.08

Every result is expressed as value ± SEM. T represents wrist temperature results, A motor activity, P body position. IS refers to interdaily stability, IV to intradaily variability, RA to relative amplitude, L5 to the time when the minimum 5 hours average for every variable is found, M10 to the time when the maximum 10 hours average is found for every variable. VL5 and VM10 refer to every variable value for L5 and M10. All variables except L5 and M10 are expressed in arbitrary units.

The time of day (within a period of 5 consecutive hours) when the variables presented the lowest values (L5) was very similar for all of them. As expected, the midpoint of L5 took place during the night, between 04:10 (for A) and 04:49 h (for P). This time can be considered as a valuable phase reference for the circadian system, and coincided with the maximum value of T and the center of the sleep period. M10, the midpoint of the 10 consecutive hour period (when the variable presents maximum values) and a phase marker for the temporal location of the center of the activity period, occurred at mid-day, although with wide dispersion among the different variables (from 15:09 h for P to 17:21 h for T).

**Figure 4:**
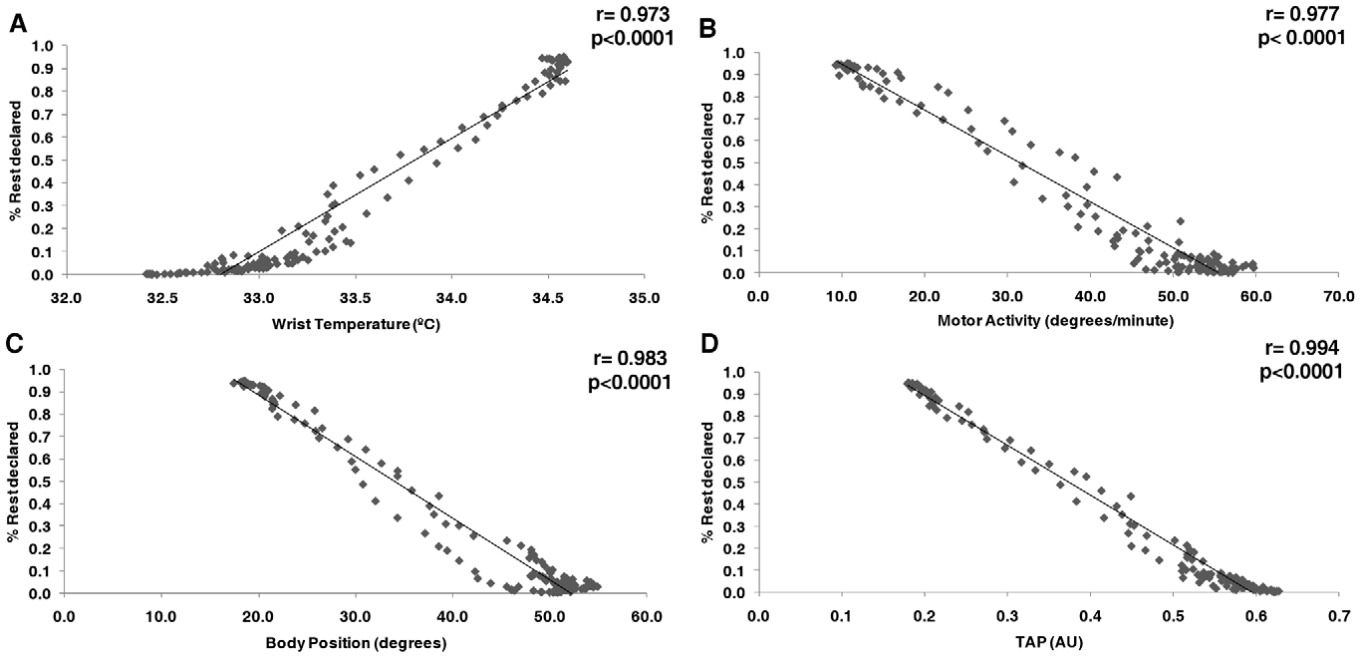
Correlations between every variable with respect to rest declared by subjects. Wrist temperature (A), motor activity (B), body position (C) and TAP (D). Please note correlations coefficients and its probability value on the upper right of every pannel.

In order to objectively evaluate whether TAP improved the accuracy of rest-activity prediction with respect to each variable considered separately, we analyzed the correlation between rest probability, as reported by the subjects in their logs, and temperature, motor activity and body position (Figure 4). Sleep logs reliability was assessed by checking diaries' compliance. We found that an 81% of these logs contained all the information asked and were filled in correctly for the whole study. A positive correlation between wrist temperature and sleep as reported by the subjects proved to be both very strong and significant (r = 0.973, p<0.0001). This correlation was even stronger, albeit now negative, for activity (r = −0.981, p<0.0001), position (r = −0.983, p<0.0001) and TAP, this latter reaching the maximum value for the correlation coefficient, (r = −0.993, p<0.0001). This improvement for rest-activity deduction by TAP proved to be significantly higher when compared with temperature (p = 0.0003), activity (p = 0.0012) and position (p = 0.0121).

In order to determine whether TAP is a reliable marker capable of accurately predicting rest-activity periods, we calculated agreement rates and specificity and sensitivity tests comparing the prediction from each variable (including TAP) and the rest periods reported by the subjects (Table 2). Again, agreement rates, as well as specificity and sensitivity results, reached their highest values for TAP. TAP's scores in sensitivity, specificity and agreement rates were significantly different from each single variable (except for body position in the sensitivity test), proving again that TAP describes more accurately rest-activity rhythm than separate variables (Table 3).

**Table 2:** Sensitivity, specificity and agreement rates results.

	Sensitivity	Specificity	Agreement rates
**T**	0.5500	0.7600	0.8160
**A**	0.5800	0.7700	0.8261
**P**	0.6300	0.7900	0.8425
**TAP**	0.7400	0.8700	0.9043

T refers to wrist temperature, A to motor activity, P to body position and TAP to the integrated variable. For further information in statistical differences among these parameters, please see tables of contingency in the supporting file information's section.

**Table 3:** Contingency tables, chi squared and p values for sensitivity, specificity and agreement rates results presented in Table 2.

	Sensitivity		Specificity		Agreement rates	
	χ2 = 1680.330 (p<0.00005)		χ2 = 1419.116 (p<0.00005)		χ2 = 1414.011 (p<0.00005)	
**T**	10054	8372+	27064	8372+	37118	11699+
**A**	11014	7912+	26564	7912+	37578	11239+
**P**	11991	7166	26333	7166+	38324	10493+
**TAP**	12776+	4388	28669+	4388	41445+	7372

T refers to wrist temperature, A to motor activity, P to body position and TAP to the integrated variable, (see table 2 for more information).

**Figure 5:**
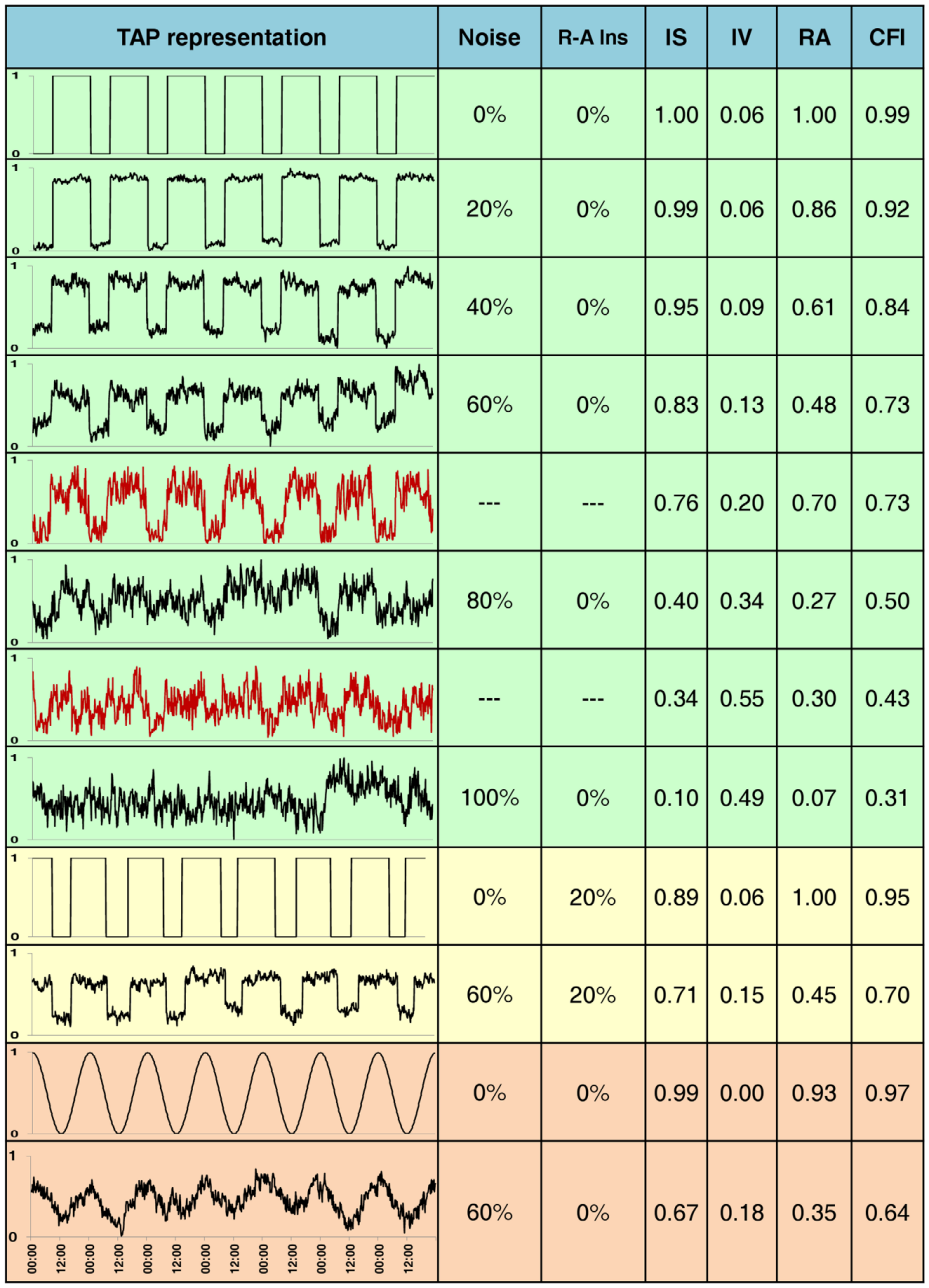
TAP simulations. On the first column, TAP patterns obtained from simulations performed on Syntesi (Diez-Noguera, Barcelona, 2007) are shown. These simulations are drawn on black and subject's real TAP pattern on red. The noise percentage employed for modelisation (% noise) and percentage of instability in rest-activity ratio (R-A Ins) are shown in the second and third columns, respectively. Calculations of interdaily stability (IS), intradaily variability (IV), relative amplitude (RA) and circadian function index (CFI) for every model or real case are also indicated. Note that IS, IV, RA and CFI's units are AU. Green shadow indicates simulations performed on squared waves under different noise levels (while the same R-A Inst is maintained) and subject's real TAP pattern. Yellow shadow indicates simulations performed on squared waves under a 20% of R-A Inst and two extreme noise levels. Orange shadow indicates simulations performed on sinusoidal waves under two different noise levels (while the same R-A Inst is maintained).

One step further into the study of the human circadian system by means of non-invasive techniques was to devise a new quantitative Circadian Function Index (CFI), consisting of three parameters calculated based on the TAP non-parametric analysis. CFI scores were then calculated for all subjects. Furthermore, all simulated TAP series were also subjected to non-parametric analysis and CFI calculation. Results are shown in figure 5, with both real and simulated TAP values. It can be seen how increasing levels of noise had the effect of lowering IS and RA values and increasing IV levels. A close inverse relationship was observed between CFI and the noise level in the simulated TAP. When introducing 20% of instability in rest-activity ratio for two levels of noise (0 and 60%), we can observe how CFI values decrease when the instability is introduced, both under the 0 and 60% of noise conditions.

TAP patterns, non-parametrical analyses and CFI for two real subjects (scoring the highest and the lowest CFI, respectively) have been inserted in the figure between the simulations. CFI scores perfectly matched TAP waveforms, with CFI values being inversely related to the noise level. When focusing on the real subject with CFI = 0.73, we can appreciate how his/her CFI coincided with the 60% noise simulation. IS and IV values were similar in both cases, whereas RA was much higher for the subject. Small differences were found between the second real subject and the 80% noise simulation. However, the graphic representations for both matched quite closely.

As can be seen from this figure, the real subjects scored CFIs between 0.73 (best) and 0.43 (worst). This shows a narrow band within which a homogeneous real population should lie.

Also, we wanted to know whether CFI behaved as expected for other types of waveforms and so we performed these simulations on sinusoidal waves concerning only noise quantity. Again, CFI decreases when the percentage of noise increases, proving that it is very sensitive to circadian disturbances in the waveforms tested.

Finally, we performed correlations between CFI and two parametrical tests that assumed a sinusoidal adjustment: power content of the first harmonic and percentage of variance explained by the rhythm in a cosinor analysis. We only found a strong correlation between CFI and first harmonic (r = 0.532, p<0.001). Correlation between CFI and percentage of variance was r = 0.014, p = 0.919).

## Discussion

Circadian chronodisruption constitutes a heterogeneous group of circadian system impairments, but no objective examination methods are currently being used for their clinical diagnosis in humans. Thus, a new quantitative strategy for assessing circadian system status will facilitate the development of clinical applications of Chronobiology. The present study describes for the first time a new variable, TAP, which integrates the simultaneous information from: wrist temperature, motor activity and body position, in addition to the implementation of a quantitative index for scoring circadian robustness (CFI). TAP provides a reliable and accurate assessment of human circadian system status and the ability to detect rest-activity cycles in large populations under ambulatory conditions.

We selected wrist temperature as part of our TAP variable because it is the result of internal and external influences and provides integrated information about the master pacemaker function and internal and external *zeitgebers*. In our study, high wrist temperature is closely linked to sleepiness, probably through parasympathetic activation and skin blood vessels vasodilation, while it drops during arousal periods, associated with sympathetic activation and vasoconstriction [23]. The temperature rhythm minimum, occurring between 20:00–22:00h, a period previously known as “wake maintenance zone” that coincides with the start of the nocturnal melatonin surge, dim light melatonin onset (DLMO) [24], and could be used as a reliable phase marker for circadian system timing. In spite of this, however, wrist temperature is the single variable that showed the lowest correlation, agreement rates, sensitivity and specificity values for rest-activity prediction, probably because of its endogenous component, which makes it more difficult to be modified by the subject's voluntary activity than other variables such as motor activity and body position. This endogenous character can also be inferred from the existence of a temperature increase anticipation prior to sleep time, as described in the results.

Actimetry has been proposed as a substitute for other complex, expensive methodologies such as polysomnography or core body temperature to evaluate circadian system status in humans [11]. A number of studies with patients support the importance of a robust day-night rhythm in order to remain healthy. For example, in patients with metastatic colorectal cancer, a marked rest-activity rhythm as recorded by actigraphy was associated with better quality of life and better survival rates [25].

Furthermore, to the best of our knowledge, body position has never been used in the assessment of human circadian functionality, mainly because most actimeters are placed on the wrist. Since we placed the actimeter aligned with the arm, we were able to differentiate between when the subject is in and out of bed. Analyzed separately, motor activity and body position showed higher correlations and agreement rates (as well as specificity and sensitivity values) with the rest-activity periods reported by subjects than wrist temperature did. This is not surprising, considering that both variables respond to sleep and their values vary dramatically at the exact moment of waking up or lying down. These variables are less dependent on the endogenous component of the circadian system than the wrist temperature rhythm.

However, each variable individually introduces specific artifacts, as being influenced by several external signals. Thus, the use of an integrative variable combining the study of several variables allows to correct mistakes attributable to the interpretation of single variables. For example, the masking effects of sleep on temperature can be eliminated by taking into account motor activity [26]. It is true that when averaging T, A and P, we can occasionally lose information from isolated variables. Nevertheless, TAP does not exclude the possibility of parallel single variable analysis in some cases. Then, if we needed to determine the precise time to go to bed, body position analysis by itself would be useful. If, in addition, temperature is high and activity is low, it would indicate that the subject is not only lying, but sleeping.

When considering non-parametric analysis, it should be stressed that the timing of L5 was very stable for all variables studied. It took place around 4 AM, during the second half of the night, when REM sleep (characterized by minimum muscle tone [27], [28]), is more likely to occur and melatonin reaches its nocturnal peak [29]. This was the time when the greatest homogeneity among subjects was found.

Despite the fact that motor activity and body position showed good rates of agreement and a high correlation with rest as reported by the subjects, these results improved even further for the integrated TAP variable (with an agreement rate of 90.43% and r = 0.993), which would indicate that TAP is able to deduct rest-activity periods more reliably than any of the separate variables by themselves. As also expected, sensitivity and specificity results were better for TAP than for single variables. Other authors have found high levels of sensitivity (the ability to detect sleep) but low levels of specificity (the ability to detect wake states) [30]–[32] when actigraphy was compared with polysomnography (PSG). This is due to the fact that in these studies, PSG was performed over one night only. During these few hours, the subjects spent most of their time sleeping, which is why the results of the sensitivity tests for actigraphy were so high. In our work, however, we found not only high levels of sensitivity (mean = 0.74), but also high levels of specificity (mean = 0.87), in spite of the fact that we expanded our analysis over a period of 7 days and considered both nocturnal and diurnal reported rest. These results indicate that our TAP variable is very reliable for detecting rest, but also very consistent for identifying wake states. This is in contrast to the results of Wang et al. [33], for example, who found a sensitivity of 0.95 and a specificity of 0.41 when comparing actimetry to PSG during a single night.

All correlation factors, agreement rates and specificity-sensitivity values were compared to the rest-activity diaries. These logs had the advantage of allowing subjects to record information at the same time that the event actually took place. However, they did not always prove to be an objective measure of sleep timing, and they were dependent upon the subject's willingness to complete them correctly [18], an 81% in our study.We are aware that one limitation of our study is the fact that PSG was not used as a reference for sleep evaluation; however, our main objective was not merely to determine sleep parameters, but rather to evaluate the circadian system status in free-living subjects during a representative period of their lives. In this regard, it has been suggested that PSG may not be the best method to which subjective sleep evaluation tools should be compared [34]. Nevertheless agreement rates as ours, varying from 88% to 97%, have been described [35].

Another factor to consider is that in order to improve the ability of actigraphy to predict sleep periods, most commercial brands of actimeters have developed complex algorithms adapted to a specific population; however, the use of highly specific algorithms impairs sleep detection when studying other age groups or patients with different medical conditions, making inter-group comparisons very difficult. For example, a decrease in the accuracy of actimetry for sleep detection was seen in a study of patients with neurological and other medical conditions associated with ageing [17]. In addition, it has also been suggested that actigraphy needs further improvement in order to accurately evaluate sleep-wake cycles in newborns [36].

The TAP variable allows us to predict rest-activity periods very accurately. In this sense, we strongly believe that our method allow us getting rid of sleep logs, subjected to volunteers' degree of compliance.

Dichotomy indexes such as I<O, I>O and autocorrelation indexes used as indicators of the circadian system status have been successfully correlated to pathological states such as cognitive deficits [4], colorectal cancer outcomes [26] and shift work [11]. However, these indexes only provide partial information about the rest-activity ratio in and out of bed, and exclude other significant sources of information. Therefore, we decided to create an index, CFI, based on three circadian parameters, each one providing complementary information about the circadian system. Interdaily stability [37] indicates the regularity of the day-to-day TAP pattern. Intradaily variability, a measure of the fragmentation of the rest-activity rhythm, is more dependent on endogenous circadian disturbances. It shows a moderate correlation with functional, social and emotional well-being. For example, fragmentation increases in association with dementia, cognitive deficits, etc [37]. Finally, amplitude is the result of both internal and external influences. Amplitude is high in subjects with a healthy circadian system and who have a stable daily routine. CFI allows us to classify the circadian system status of a population according to the overall TAP rhythm.

CFI proved to be very accurate when trying to define the subjects' circadian status and responds as expected (decreasing) when instability and noise of the rest-activity ratio increases. Next step in the study of TAP will imply populations with circadian disturbances, such as the elderly, shift workers, cancer patients,…where a reduced amplitude and higher fragmentation in their TAP rhythm would be expected.

In conclusion, our results show that the TAP variable, which combines information from temperature, actimetry and position, constitutes a step forward in the ambulatory evaluation of circadian system status in humans. TAP provides information about the status of the circadian system because it includes a variable with a large endogenous component (temperature), but also variables that are more reactive to behavioral demands, such as motor activity and body position. Furthermore, CFI allows for the quantitative classification of populations and provides important information about the circadian timing system, which facilitates the objective evaluation of the efficacy of treatments to improve chronodisruption.
